# High sensitivity of cloud formation to aerosol changes

**DOI:** 10.1038/s41561-025-01662-y

**Published:** 2025-04-03

**Authors:** Annele Virtanen, Jorma Joutsensaari, Harri Kokkola, Daniel G. Partridge, Sara Blichner, Øyvind Seland, Eemeli Holopainen, Emanuele Tovazzi, Antti Lipponen, Santtu Mikkonen, Ari Leskinen, Antti-Pekka Hyvärinen, Paul Zieger, Radovan Krejci, Annica M. L. Ekman, Ilona Riipinen, Johannes Quaas, Sami Romakkaniemi

**Affiliations:** 1 Department of Technical Physics, University of Eastern Finland, Kuopio, Finland; 2 Finnish Meteorological Institute, Kuopio, Finland; 3 Department of Mathematics and Statistics, Faculty of Environment, Science and Economy, University of Exeter, Exeter, UK; 4 Bolin Centre for Climate Research, Stockholm University, Stockholm, Sweden; 5 Department of Environmental Science, Stockholm University, Stockholm, Sweden; 6 Research and Development Department, Norwegian Meteorological Institute, Oslo, Norway; 7 Finnish Meteorological Institute, Helsinki, Finland; 8 Department of Meteorology, Stockholm University, Stockholm, Sweden; 9 Leipzig Institute for Meteorology, Leipzig University, Leipzig, Germany; 10 Present Address: Institute of Chemical Engineering Sciences, Foundation for Research and Technology-Hellas, Patras, Greece

**Keywords:** Atmospheric science, Environmental sciences, Climate change

## Abstract

The susceptibility of cloud droplet number to cloud condensation nuclei number is one of the major factors controlling the highly uncertain change in the amount of solar radiation reflected by clouds when aerosol emissions are perturbed (the radiative forcing due to aerosol–cloud interactions). We investigate this susceptibility in low-level stratiform clouds using long-term (3–10-yr) in situ observations of aerosols and clouds at three high-latitude locations. The in situ observations show higher susceptibility for low-level stratiform clouds than values reported for satellite data. We estimate −1.16 W m^−2^ for the aerosol indirect radiative forcing on the basis of our observations, which is at the higher end of satellite-derived forcing estimates and the uncertainty range of the most recent Intergovernmental Panel on Climate Change report. We evaluate four Earth system models against the observations and find large inter-model variability in the susceptibility. Our results demonstrate that, even if the susceptibility in some of the models is relatively close to observations, the underlying physics in the models is unrealistic when compared with observations. We show that the inter-model variability is driven by differences in sub-grid-scale updraught velocities and aerosol size distributions, raising a need to improve these aspects in models.

## Abstract (web summary)

In situ observations indicate a greater susceptibility of cloud droplet number to cloud condensation nuclei than is estimated from satellite observations, which suggests that aerosols exert a stronger radiative forcing than previously thought.

## Main

Radiative forcing by aerosols through aerosol–cloud interactions (RFaci) is the most important anthropogenic cooling component contributing to Earth’s radiative forcing, and it remains the largest source of uncertainty in radiative forcing estimates^1^. The magnitude of RFaci is mainly based on estimates from global climate models and satellites, both of which show a large range^1^. The susceptibility (*S*) of the cloud droplet number concentration (*N*_d_) to the cloud condensation nuclei (CCN) concentration is a major factor in determining the effect of anthropogenic aerosols on the Earth’s radiative budget, as droplet formation linking aerosols and cloud microphysics is at the heart of the first aerosol indirect effect^2–8^. According to the classical Twomey effect, an increase in the CCN in liquid clouds leads to a monotonic increase of *N*_d_ for a given updraught velocity^9^. The increase in the CCN leads to a decrease in the maximum water vapour supersaturation at a given updraught, resulting in a logarithmic relationship between *N*_d_ and CCN^3,10^. In conditions where aerosol concentrations are low or updraught velocities are high, a high susceptibility of *N*_d_ to CCN is expected. In contrast, low susceptibility can be expected in cases where the updraught velocity (or supersaturation conditions) limits the number of activated cloud droplets rather than the availability of CCN. At the extremes, these conditions are commonly referred to as aerosol- and updraught-limited regimes of cloud droplet formation^11^.

Satellite observations of *N*_d_ and various satellite-derived or reanalysis-based proxies for CCN have recently been used to constrain *S* (refs. ^7,12–15^). While satellite observations offer the only observational approach to constrain the Twomey effect at global scale, there are a number of important limitations affecting the analysis, related to both the capability of remote sensing to detect CCN concentrations at the cloud base where the droplet activation takes place as well as deriving *N*_d_ accurately^7,8,15–18^. Since both of these quantities are central for accurate constraints on *S*, there is a need to investigate *S* using in situ observations to evaluate and complement the insights from remote sensing observations.

In global climate models, the *N*_d_ response to changes in aerosol concentrations and properties depends on the cloud droplet activation parameterization, the model representation of aerosol properties and the parameterization for the sub-grid-scale updraught velocity at the cloud base^19^. Aerosol fields simulated by climate models have been evaluated against aerosol observations^20–24^, and several successful closure studies have been conducted with parcel and chemical-transport models to validate droplet activation parameterizations^25–27^. Yet, there is currently a lack of multi-model studies comparing susceptibilities from global climate models directly with long-term coinciding in situ observations of *N*_d_ and the CCN at the cloud base.

Low-level stratiform clouds are ideal to assess the Twomey effect and provide constraints on *S* because they are usually shallow, mainly non-precipitating and cover large surface areas. In this Article, we investigate the susceptibility of *N*_d_ to CCN concentrations using long-term coinciding in situ observations of clouds and aerosol particles from three observatories in the Northern Hemisphere belonging to the Aerosol, Clouds and Trace Gases Research Infrastructure (ACTRIS) (Puijo, Pallas and Zeppelin, representing semi-urban, remote and Arctic remote environments, respectively, with different aerosol and updraught characteristics^28–31^; Methods and Supplementary Fig. 1). The observations are used to evaluate four global climate models: European Center Hamburg Atmospheric Model with Hamburg Aerosol Model (ECHAM-HAM)^32,33^, ECHAM with Sectional Aerosol Module for Large Scale Applications (ECHAM-SALSA)^33,34^, Norwegian Earth System Model (NorESM)^35^ and United Kingdom Earth System Model (UKESM)^36,37^. The models differ conceptually in the way they represent the aerosol size distribution: ECHAM-SALSA uses a sectional representation, while the other models use modal representations. In all the models, the cloud droplet activation parameterizations are based on the same physical representation of droplet activation but differ in how the aerosol size distribution is represented: modal models use the Abdul-Razzak and Ghan^38^ parameterization, while SALSA uses the Abdul-Razzak and Ghan^39^ version of the parameterization. All models parameterize the updraught (*w*) at the cloud base from the turbulent kinetic energy (TKE). However, UKESM employs a full probability distribution of *w* to calculate *N*_d_, whereas the other models use a characteristic updraught (*w¯*). *w¯* is connected to the variability of *w* through the standard deviation (*σ*_*w*_), which is assumed to follow a normal distribution in stratiform clouds. In practice, it is computed by scaling with the square root of the TKE^40^. To be able to compare the models and observations as well as intercompare the models, we present all updraughts as *σ*_*w*_ (Methods).

In our efforts to constrain *S* directly from in situ observations, we use *N*_70_ (the concentration of aerosol particles larger than 70 nm in diameter) as a proxy for the CCN, corresponding to a maximum supersaturation of about 0.25–0.45% (ref. ^41^). *N*_d_ is measured using specific sampling systems designed for in-cloud studies (Methods). The results in Supplementary Fig. 2 show that the used lower size limit of the CCN proxy has only a minor effect on *S* and does not affect our conclusions, therefore justifying this choice. For each of the three locations, we use instantaneous 3-h time resolution model data from the grid box corresponding to the horizontal and vertical location of the station using nearest neighbour interpolation. Our analysis shows that there is a notable inter-model variability in the susceptibility of *N*_d_ to CCN. Furthermore, even if some of the models are in relatively good agreement with the observations, the description of the underlying physical processes and the central model parameters show large discrepancies from the observations.

## Aerosol size distributions and CCN concentrations

**Fig. 1:**
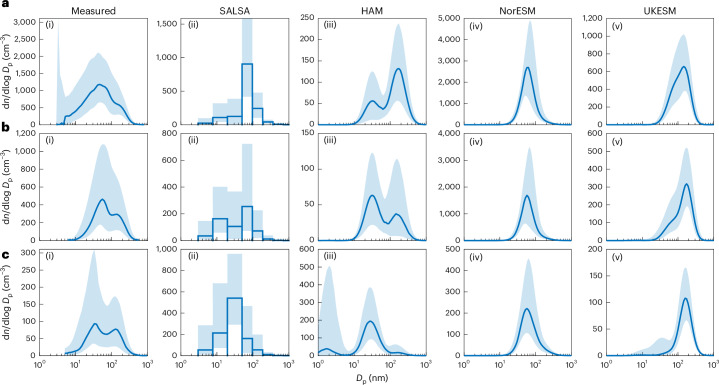
Measured and modelled median aerosol number size distributions for different locations. **a**, The measured distributions (i) and model outputs for ECHAM-SALSA (ii), ECHAM-HAM (iii), NorESM (iv) and UKESM (v) for Puijo. **b**,**c**, Corresponding measured distributions and model outputs for Pallas (**b**) and Zeppelin (**c**). Note the different *y*-axis scales. The shaded areas indicate the 25–75th percentile ranges. *D*_p_ is the particle diameter. Source data

As the number of CCN is highly dependent on the aerosol size distribution characteristics, we show the measured and modelled number median size distributions at the three measurement sites in Fig. 1. Bimodal median size distributions are observed at all sites (Fig. 1a(i)–c(i)). In terms of the CCN concentration (that is, *N*_70_), Puijo shows the highest concentrations and Zeppelin the lowest, with approximately an order of magnitude difference in the median concentration (Supplementary Fig. 3). The models display a large inter-model variability in the median size distributions. It is notable that both ECHAM versions and UKESM show bimodal distributions in most cases, while NorESM shows unimodal distributions. In general, ECHAM-HAM has lower CCN concentrations compared with observations at all three locations, while NorESM and UKESM do not capture the lowest concentrations at Puijo and Pallas (Fig. 1 and Supplementary Fig. 3).

## Susceptibility of *N*_d_ to CCN

**Fig. 2:**
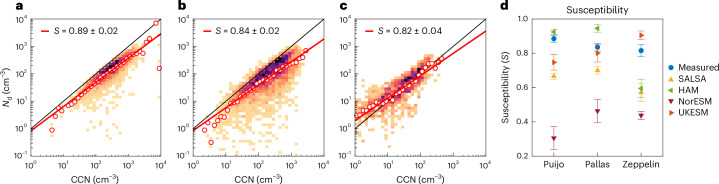
Measured *N*_d_–CCN joint histograms and *S* for Puijo, Pallas and Zeppelin. **a**–**c**, Joint histograms for Puijo (**a**), Pallas (**b**) and Zeppelin (**c**). The colour indicates the probability (orange, low; black, high), and red circles show mean *N*_d_ values for a certain CCN bin. **d**, The fitted value for *S* defined for the observations and the model output at the three locations. Error bars represent 95% confidence bounds, and the number of data points (*N*) for each fit is given in Fig. 3 and Supplementary Figs. 5 and 6. The black line is the 1:1 line. Source data

To investigate the susceptibility *S*, we use the joint histogram approach^4,5^. *S* is defined through an ordinary least-squares (OLS) fit to the logarithmic *N*_d_–CCN data points. Figure 2a–c shows the measured joint histograms for Puijo, Pallas and Zeppelin. The observation-based susceptibilities at all three stations vary between 0.82 and 0.89. Note that the size limit of the used CCN proxy partly defines the position of the data points with respect to the 1:1 line in the joint histograms: if the size limit of the chosen proxy is notably higher than the lowest size of the activated aerosol, a large fraction of the data points are above the 1:1 line. Hence, Fig. 2c indicates that, at Zeppelin, the activation diameter is smaller than 70 nm in many cases^42^.

**Fig. 3:**
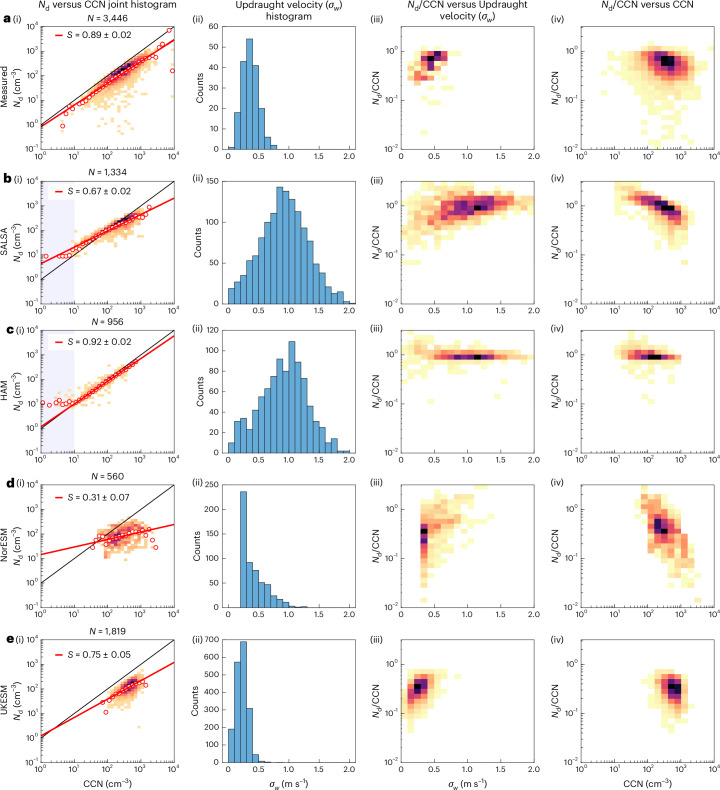
Joint histograms and updraught velocities for Puijo. **a**, Measurements of (i) the *N*_d_–CCN joint histograms with corresponding *S* values (and 95% confidence bounds), where the colour indicates probability (orange, low; black, high) and red circles show mean *N*_d_ values for a certain CCN bin and *N* indicates the number of data points; (ii) the measured and modelled p.d.f. for *σ*_*w*_; the ratio of *N*_d_ to CCN plotted as a function of (iii) *σ*_*w*_ and CCN (iv) concentration, where the colour indicates probability intensity (orange, low; black, high). **b**–**e**, Corresponding results from the SALSA (**b**), HAM (**c**), NorESM (**d**) and UKESM (**e**) models. The set value of 10 cm^−3^ used as the lower limit for *N*_d_ is marked by the shaded area in **b**(i) and **c**(i). Source data

When the models are compared with each other and the measurements, a large variation in *S* can be seen (Fig. 2d). In general, NorESM gives notably lower values (0.31–0.46) than the other models. For Puijo and Pallas, ECHAM-HAM yields *S* values that are slightly higher than the measurements (0.93–0.95), while the ECHAM-SALSA version gives lower susceptibilities than the observations (0.67–0.70). For the high Arctic location, UKESM shows the highest sensitivity of *N*_d_ to CCN (0.91). Note that the two ECHAM model versions have a set value of 10 cm^−3^ as the lower limit of *N*_d_ (Fig. 3b(i),c(i), shaded area), hence the shaded area is omitted when calculating *S*. In NorESM, *N*_d_ is not limited, and in UKESM, the lower limit of *N*_d_ is low enough (5 cm^–3^) not to affect the analysis.

The activation, and hence *S*, is controlled by the CCN concentrations and updraughts (which govern the supersaturation conditions at the cloud base). Hence, to understand the differences in *S* between the observations and models, as well as the inter-model variability, we next investigated *σ*_*w*_ from observations and the model outputs.

The measured values for *σ*_*w*_ are comparable at Puijo and Pallas (Fig. 3a–e(ii) and Supplementary Fig. 5). At Zeppelin, the measured *σ*_*w*_ values are clearly higher than at other locations (Supplementary Fig. 6). Note that, at Zeppelin, the orography affects the updraughts measured at the station. This is shown in Supplementary Fig. 6a2, where there is a clear shift of *σ*_*w*_ to lower values when the investigation is limited to low horizontal wind speeds. Hence, when the modelled *σ*_*w*_, representing a larger grid box area, is compared with the observations at Zeppelin, the comparison should be done using observations limited to low horizontal wind speeds.

The models generally struggle to reproduce the observed updraught values. Both ECHAM versions give unrealistically high *σ*_*w*_ values at Puijo and Pallas, while *σ*_*w*_ peaks at too low values at Zeppelin. In NorESM, most values are around 0.2 m s^−1^ (for all three locations), which is the lower limit of *σ*_*w*_ set in the model. UKESM gives the most realistic *σ*_*w*_ probability density function (p.d.f.) for all three stations compared with observations.

## Drivers of inter-model variability in susceptibility

To understand the factors controlling *S* in the models, we first compare the modal and sectional versions of the ECHAM model (that is, ECHAM-HAM and ECHAM-SALSA, correspondingly) as both models have comparable *σ*_*w*_ p.d.f. but clear differences in *S*. Figure 3b(i),c(i) shows that, for Puijo (and Pallas; Supplementary Fig. 5), ECHAM-HAM gives clearly higher *S* than ECHAM-SALSA. To understand the modelled behaviour better, we plotted log(*N*_d_/CCN) as a function of parameters controlling this ratio, that is, *σ*_*w*_ and CCN (Fig. 3a–e(iii,iv)). In observations, and in ECHAM-SALSA, *N*_d_/CCN increases slightly with increasing *σ*_*w*_ and decreases with increasing CCN concentrations. This is the physical behaviour that can be expected for the cloud droplet activation process: the supersaturation increases with increasing *σ*_*w*_, enabling the activation of smaller particles, while increasing CCN concentration causes a faster depletion of water vapour and hence a decrease of the supersaturation, which in turn decreases the *N*_d_/CCN ratio. ECHAM-HAM, on the other hand, produces a seemingly physically unrealistic behaviour: the ratio of *N*_d_ to CCN is insensitive to both *σ*_*w*_ and CCN, indicating that the changes in supersaturation (which should induce a change in the activation diameter) do not have an apparent effect on *N*_d_. The cloud parcel model results (Supplementary Fig. 4) demonstrate that the behaviour is indeed driven by activation process, not coagulation–coalescence or entrainment processes following the activation.

**Fig. 4:**
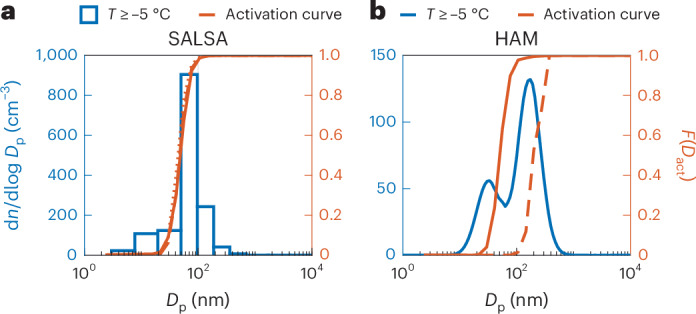
Median size distributions and cumulative p.d.f.s of activation dry diameters for the two ECHAM model versions for the Puijo location. **a**,**b**, The CHAM-SALSA outputs together with the cumulative p.d.f.s (*F*(*D*_act_)) of critical diameters (*D*_act_) of three size bins (bins 3–5) (**a**) and the ECHAM-HAM outputs together with the cumulative p.d.f. for the accumulation mode (solid line) and Aitken mode (dashed line) (**b**). Source data

Figure 4a,b shows the cumulative p.d.f.s of the minimum dry diameters required for cloud activation for ECHAM-SALSA and ECHAM-HAM. For ECHAM-SALSA, these p.d.f.s are calculated for each size bin separately (the p.d.f.s for the size bins relevant for activation are shown in Fig. 4a). For ECHAM-HAM, p.d.f.s are calculated separately for each mode, as modes are activated separately in HAM. In HAM, the activation dry diameters (the dry size of the smallest aerosols that are activated) are very low for the accumulation mode owing to the high *σ*_*w*_ (Fig. 4b). Hence, in most cases, the entire accumulation mode is activated. On the other hand, when we investigate the Aitken mode, the activation diameters are very large and clearly outside of the mode size range. This model behaviour is driven by differences in the chemical composition of the Aitken and accumulation modes accompanied by simplifications made in the activation calculations. First, diagnosing the simulated composition of different modes shows that, in ECHAM-HAM, the Aitken mode contains a smaller fraction of soluble material than the accumulation mode, resulting in lower hygroscopicity. This shifts the activation diameter of Aitken mode particles to larger sizes compared with the accumulation mode. Second, in modal models, the Kelvin term of the Köhler equation^38^ is calculated using the geometric number mean diameter of the mode. This simplification further shifts the activation diameter of the Aitken mode to larger sizes, while it has a minor effect on the activation diameter of the accumulation mode. The effect is enhanced when the aerosol hygroscopicity is low. Taken together, the behaviour of ECHAM-HAM shown in Fig. 3c(iii,iv) and Supplementary Figs. 5 and 6 is an interplay of high *σ*_*w*_, the shape of the size distributions, too low CCN concentration and Aitken mode hygroscopicity, and simplifications in calculating the activation. In ECHAM-SALSA, the median size distributions for Puijo and Pallas have a maximum coinciding with the activation limit (Fig. 4a and Supplementary Fig. 7a), making *N*_d_/CCN sensitive to changes in the supersaturation. In contrast, *N*_d_/CCN in ECHAM-HAM is insensitive to changes in supersaturation, as the whole accumulation mode is above the activation diameter while the Aitken mode is too small to be activated (Fig. 4b and Supplementary Fig. 7b). A different behaviour is observed for ECHAM-HAM at Zeppelin, where the Aitken mode overlaps with the p.d.f. of critical diameters (Supplementary Fig. 8), making the model sensitive to changes in supersaturation and supporting our hypothesis.

In the case of UKESM and NorESM, the narrow simulated range of CCN values at Puijo and Pallas (Supplementary Fig. 3) limits the definition of *S*: the lower CCN concentrations are not represented in either model for Puijo and Pallas. UKESM gives *σ*_*w*_ p.d.f.s closest to observations, and also *S* is reasonable compared with observations at all locations. NorESM gives systematically too low *S* at all three locations. This could be due to the *σ*_*w*_ p.d.f. being dominated by the lowest *σ*_*w*_ bin in NorESM, which could on its own result in an updraught limited regime. In the case of NorESM, the critical activation dry diameters (approximately 100–400 nm) usually fall in a strongly decreasing region in the size distribution (on the decreasing side of a fairly large Aitken mode). Hence, the increase in the activation diameter caused by the maximum supersaturation suppression with increasing CCN results in a stronger reduction in activated aerosol compared with models where the critical activation diameter falls in a size range where the number concentration is constant or increases with size. This makes *N*_d_ in NorESM less sensitive to increasing CCN compared with the other three models in the studied conditions. This is consistent with some previous studies^12,43,44^.

## Implications for RFaci estimates

Our analysis of in situ observations shows higher susceptibility of *N*_d_ to CCN for low-level stratiform clouds (0.82–0.89, depending on the site) than values reported for satellite data (approximately 0.3–0.8)^5,12^. This is supported by some earlier work^6,45–47^. The difference can be partly due to different cloud regimes and partly due to differences in the methodology: ground-based in situ observations define *N*_d_ and CCN at the cloud base, while satellite-based estimates are representative for *N*_d_ at the cloud top and use different proxies for the CCN column number^5^. In addition, at many locations, the size ranges that cause the major variability in the CCN concentrations are too small to be detected by remote sensing methodologies, highlighting the relevance of in situ observations to constrain *S*. Our in situ observations using long-term data on collocated CCN and *N*_d_ measurements represent the higher end of previous in situ-based estimates of *S*, while many of the previous estimates are based on aircraft measurements where CCN and *N*_d_ are non-collocated in time and space^48–50^, complicating the data interpretation. *S* values derived using CCN proxies retrieved from satellite polarimetry^5^ are consistent with ours in the relevant regions. However, it is unclear whether the satellite-based estimate is reliable, since it contains systematic biases of both sign^18^. A value of 0.8 for the susceptibility was used by Bellouin et al. to estimate the upper range of RFaci on the basis of satellite and in situ observations^6,12^. If we use the mean of our observed values and follow the method of Bellouin et al., we get an estimation of −1.16 W m^−2^ for the upper range of RFaci. This may still be a conservative estimate since the framework of Bellouin et al. makes use of the aerosol optical depth to quantify the aerosol, which is known to lead to an underestimation of RFaci^4^. The value estimated on the basis of our results still is at the higher end of the uncertainty range of the last Intergovernmental Panel on Climate Change report (−0.7 ± 0.5 W m^−2^)^51^ and satellite-derived forcing estimates (−1.1 to −1.0 W m^−2^)^5,12^.

In the four climate models investigated, *S* varied by a factor of 2–3. This variation generates substantial uncertainty in the forcing estimates when considering that the stratocumulus clouds cover a fifth of the Earth’s surface^52^. It is important to highlight that this study focuses on the most studied cloud type, where solid cloud deck covers large areas with small spatial variability, and on the simple droplet activation process, while more complicated cloud adjustments are not considered. Regardless of this, the models have clear problems in describing the susceptibility of *N*_d_ to CCN correctly.

Our results demonstrate that, even if *S* in some of the models was relatively close to observations, the underlying physics in the models is unrealistic when compared with observations. Furthermore, we demonstrate that the model difference in *S* is driven mainly by problems in defining the updraughts and the aerosol number size distributions. As there was a large difference also between the modal models using the same activation parameterization^38^, we can conclude that the cloud droplet activation parameterization does not per se cause the difference. However, the simplifications made when calculating the activation may enhance problems, for example, arising from unrealistic updraught values. Our results highlight the importance of an interplay between the main factors driving the variability, that is, how the problems in the aerosol size distribution combined with unrealistic updraught velocities may lead to too low or high susceptibilities. Hence, substantial improvements in the representation of aerosol size distributions as well as updraught velocities are needed to represent the Twomey effect correctly in large-scale models. As the effective radiative forcing due to aerosol–cloud interactions scales with *S* (refs. ^6,53^), there is an obvious need to improve the observational constraints of these factors controlling the susceptibility by using both in situ and remote sensing techniques. These improvements are the key to reduce the uncertainties on aerosol indirect forcing estimates in climate models.

## Methods

In this study, we use *N*_70_ (the concentration of aerosol particles larger than 70 nm in diameter) as a proxy for the CCN. For Salsa, *N*_70_ is calculated from

$${N}_{70}=\mathop{\sum }\limits_{i=1}^{k}\min \left({n}_{i},\max \left(0,{n}_{i}\left(\frac{\uppi }{6}{D}_{i,\mathrm{hi}}^{3}-\frac{\uppi }{6}{D}_{0}^{3}\right) / \left(\frac{\uppi }{6}{D}_{i,\mathrm{hi}}^{3}-\frac{\uppi }{6}{D}_{i,\mathrm{lo}}^{3}\right)\right)\right),$$

where *D*_0_ = 70 nm, *n*_*i*_ is the number concentration in size section *i*, *D*_*i*,hi_ is the upper limit of size section *i, D*_*i*,hi_ is the lower limit of size section *i* and *k* is the number of size sections. For modal models, *N*_70_ is calculated from

$${N}_{70}=\mathop{\sum }\limits_{i=1}^{k}{n}_{i}\frac{1}{2}\left(1-\mathrm{erf}\left(\frac{\mathrm{ln}\left(\frac{{D}_{0}}{{D}_{\mathrm{g},i}}\right)}{\sqrt{\mathrm{ln}\,{\sigma }_{\mathrm{g},i}}}\right)\right),$$

where *D*_0_ = 70 nm, *n*_*i*_ is the number concentration in mode *i*, *k* is the number of modes, *D*_g,*i*_ is the geometric mean diameter for mode *i*, *σ*_g,*i*_ is the geometric standard deviation for mode *i* and erf(·) is the error function.

We limit our analysis to liquid clouds by using a temperature limit of *T* ≥ −5 °C, for both the observational and model data. No sorting for precipitation was done. To be able to compare models and observation as well as intercompare models, we present all updraughts as standard deviations (*σ*_*w*_). *σ*_*w*_ is calculated only for the cloudy periods, and for models, *σ*_*w*_ corresponds to the conditions at the cloud base.

### In situ observations

For in situ observations, 1-h averages are calculated for the investigated parameters, both to decrease the variability caused by aerosol sampling methods and to make sure that the sampled clouds are stratified with continuous coverage, thus enabling consistent comparison with global model data. The measurement system for aerosol size distributions and *N*_d_ (dual-inlet method)^54^ was comparable for Puijo and Pallas, while at Zeppelin, a different approach (the counterflow virtual impactor (CVI) inlet method^42^) was used. The cases when the stations were in cloud were verified by visibility measurements and by the sampling system itself: cloud droplets will be measured by the inlet system, and CVI only if the inlets are in cloud. Both of the used approaches are ACTRIS approved methods. The details of the measurements for each station are described below.

The representativeness of the in situ observations for a model grid cell is a prevailing and relevant question. When considering the aerosol size distributions and CCN concentrations, Puijo station and Pallas station represent environments with no strong local sources that are untypical for the surrounding area on the grid box scale. Puijo is located at semi-urban environments where the aerosol represents a mixture of biogenic and anthropogenic aerosols. The aerosol measured at Puijo tower is dominated by biogenic aerosol from surrounding forests and long range-transported aerosol, while local anthropogenic sources play a minor role^55^. In addition, the conditions in the grid box scale surroundings of the station are comparable to conditions at the station (that is, large forest and lake areas with minor local anthropogenic sources). Pallas station is located in an Arctic remote area with large areas of comparable environmental conditions^56^. As the Pallas area has no notable local or regional air pollution sources, it is deemed to provide an excellent location for the monitoring of the background air composition in Northern Europe^30^. Similarly, the Zeppelin observatory, located in the high Arctic on Svalbard, is not influenced by any local anthropogenic emissions either^31^. The aerosol size distributions follow a very typical seasonal cycle^29^, as do the long-term cloud residual size distribution^42^ and also cloud residual black carbon concentrations from anthropogenic and long range-transported sources^57^, both of which are not notably influenced by orographic effects, making them suitable for model validation exercises. Related to cloud properties, the requirement of continuous cloud sampling is 1 h, thus ensuring we are analysing continuous cloud decks and averaging out small scale variability in the cloud properties that could not be resolved with large scale models. When the updraughts are considered, they are measured by Doppler lidar at Puijo and Pallas without considerable orographic effects. At Zeppelin, on the other hand, the updraughts are measured at the top of the hill, and the measured updraughts are affected by the orography, as shown in Supplementary Fig. 6a2, making the comparison of measured and modelled updraughts more complicated (also discussed in the ‘Susceptibility of *N*_d_ to CCN’ section).

As described below, the time periods of models and observations vary. The models are rather robust for short-term changes, especially as there are no sudden changes in input emissions or meteorology. The observations, in turn, could be sensitive to stronger annual variation. Anyhow, earlier analysis shows that, at these three sites, there is only a very weak trend in aerosol size distribution characteristics over a long time period^20^. To test the sensitivity of our analysis to chosen years, we analysed whether removing any of the years from the data sets applied for the calculation of *S* affects our analysis. The test showed that the variation in *S* is small and does not affect our conclusions.

#### Puijo

Long-term data measured at Puijo between 20 November 2009 and 31 December 2015 were used to define the CCN and *N*_d_. Size distributions of total aerosol (wet diameter cut-off of 40 µm) and interstitial aerosol (wet diameter cut-off of 1.0 µm) were measured using a two-inlet and a valve system to derive CCN and *N*_d_ concentrations^54^. *N*_d_ concentrations are calculated as the difference of the concentrations at these two inlets. Aerosol size distributions from 7 to 800 nm (3 to 800 nm after 1 February 2012) were measured with a twin differential mobility particle sizer (twin-DMPS)^58^. In this set-up, two independent DMPSs measured the particle diameters from 7 to 49 nm (3 to 53 nm after 1 February 2012) and from 30 to 800 nm with aerosol-to-sheath flow ratios of 1.4:23 and 1:5.5, respectively. The total measurement time for the total and interstitial population was 12 min. Full data inversion was applied to the raw data, including corrections for sampling losses, multiple charging probabilities, instrumental transfer functions and particle counting efficiencies, as recommended in ref. ^59^.

The vertical wind velocity was measured by using Doppler lidar (Halo Photonics) as described by Tucker et al.^60^, with 3 s time resolution for the period from 1 September 2020 to 30 November 2020. *w* measurements were carried out during the autumn season (September to November) when a large fraction of low-level liquid cloud cases occur at the Puijo location^61^. Hence, regardless of the limited data period, the representativeness of the *w* observations is good for the investigated period. To obtain *σ*_*w*_, a normal distribution was fitted to *w* observations over 1-h periods to keep the analysis consistent with aerosol and cloud observations. The *N*_d_ and CCN data used in Fig. 3a(iii) are for the same time period (1 September 2020 to 30 November 2020) and were measured as described above.

#### Pallas

The long-term data measured from 12 August 2005 to 25 December 2015 were used for Pallas station to define the CCN and *N*_d_. Similar to Puijo, a two-inlet system was used to measure the total aerosol and interstitial aerosol, and the *N*_d_ concentrations were calculated as the difference of these two (that is, in the same way as at Puijo). For the total aerosol, the design is similar to Puijo, whereas for the interstitial aerosol, the inlet design gives a wet diameter cut-off of 7 µm (ref. ^62^). On the basis of measured cloud droplet size distribution at the site^63^, the median volume diameter ranges between 9 and 19 µm. This implies that, at high CCN concentrations (low cloud droplet diameter), the observed *N*_d_ may be slightly underestimated. Aerosol size distributions were measured with identical DMPS systems from 7 to 500 nm. An aerosol-to-sheath flow ratio of 1:10 was used, and the total time resolution to obtain a size spectrum was 5.5 min. The same inversion procedure as for the Puijo DMPS data was used.

The vertical wind velocity was measured from 23 August 2022 to 15 December 2023 using Doppler lidar (Halo Photonics) as described by Tucker et al.^60^ with 11 s time resolution. The *N*_d_ and CCN data used in Supplementary Fig. 5a3 is for the same time period (23 August 2022 to 15 December 2023) and were measured as described above.

#### Zeppelin

The long-term observational aerosol–cloud in situ data measured from 27 November 2015 to 4 February 2018 at Zeppelin Observatory, Svalbard, were used to define CCN and *N*_d_ (ref. ^64^). *N*_d_ was taken from measurements of a ground-based counterflow virtual impactor inlet (GCVI, model 1205; Brechtel Manufacturing) coupled to a DMPS (custom made), which measured the cloud residual number concentration of dried cloud droplets. Only values with visibilities below 1 km and ambient temperatures above −5 °C were used as cloud data. The measurements by the GCVI were evaluated by co-located cloud droplet measurements and compared well for liquid clouds. The CCN (that is, *N*_70_) data were taken from size distribution measurements that were recorded in parallel by a twinDMPS (custom-made) sampling behind a whole-air inlet. An ultrasonic anemometer (uSonic-3 Omni; METEK) located close to the inlets at Zeppelin Observatory measured the wind components and virtual temperature at a frequency of 1 Hz. More details on the set-up can be found in ref. ^42^.

### Global models

The model runs are made with 3-h time resolution diagnostics (output provided as instantaneous model values). We use size distribution and cloud property data (cloud fraction (CF), *N*_d_*, σ*_*w*_ *or w*^*−*^) at the cloud base of the model grid box corresponding to the horizontal and vertical location of the stations using nearest neighbour interpolation. The time periods for the model runs were 1 January 2009 to 31 December 2014 for ECHAM-SALSA and UKESM, 1 January 2009 to 31 December 2013 for ECHAM-HAM7 and 1 January 2009 to 31 December 2010 for NorESM. For model data, the CF limit for cloud cases is CF ≥ 0.25. General descriptions of each model are given below. The detailed model set-ups are described in Supplementary Sect. 2. The models used one characteristic updraught velocity *w*^*−*^ to represent the sub-grid-scale variability in the vertical velocity, except for UKESM, which employs the p.d.f.-based approach, assuming a Gaussian distribution of vertical velocities (*w*) across the grid box with a mean vertical velocity of *w¯* and standard deviation of *σ*_*w*_. To harmonize the observations and all the models, we present updraughts as *σ*_*w*_ for all of them by calculating *σ*_*w*_ from *w*^*−*^ according to ref. ^25^.

All models nudged their large scale meteorology towards the European Centre for Medium-Range Weather Forecasts reanalysis data ERA-Interim^65^. Nudging was done for divergence, vorticity and surface pressure in ECHAM-HAM and ECHAM-SALSA, horizontal winds and surface pressure in NorESM and horizontal winds in UKESM. For anthropogenic emissions, all models used the Community Emissions Data System^66^ and, for biomass burning, the historical global biomass burning emissions for CMIP6 (BB4CMIP)^67^. For detailed model descriptions, see the Supplementary Information.

For M7 and SALSA, we calculated the cumulative probability distributions of critical diameters for individual modes and size classes. The critical diameter *d*_crit,*i*_ for mode or bin *i* was calculated from

$${d}_{\mathrm{crit},i}={d}_{\mathrm{g},i}{\left(\frac{{S}_{\mathrm{crit},i}}{{S}_{\max }}\right)}^{2/3},$$

where *d*_g,*i*_ is the geometric mean and *S*_crit,*i*_ is the critical supersaturation of mode or bin *i*, and *S*_max_ is the maximum supersaturation^38,39^. The cumulative p.d.f.s for critical diameters are calculated from instantaneous values.

For calculating the radiative forcing in ECHAM-HAM, we used the radiative fluxes from the present-day simulation and the simulation with pre-industrial emissions. ERFaci+ari was diagnosed from the difference in the top-of-atmosphere net radiative flux between the two simulations^68^, and ERFaci from the same simulations using the approach recommended by Ghan^69^.

### Cloud parcel model

The model used to derive the theoretical number of activated cloud droplets in Supplementary Fig. 4 is an adiabatic air parcel model employing sectional representation of the aerosol size distribution. The model includes differential equations describing the condensation of water on particles of different sizes in the adiabatically cooling air parcel. All simulations are carried out in a similar manner by initializing the model at 95% relative humidity and temperature of 278 K, then raising the air parcel to the cloud with a constant updraught until a liquid water content of 0.1 g kg^−1^ is reached, and the number of cloud droplets formed is determined. For simplicity, the aerosol was assumed to be composed of 50% sulfate and 50% insoluble material, and the median aerosol number concentration from Fig. 1 was scaled to cover the range of observed and simulated aerosol concentrations.

### Defining *S* from joint histograms

The susceptibility (*S*) was determined via the OLS fitting procedure applied to the logarithmic *N*_d_–CCN joint histogram data points. The used method is comparable to the method used in a satellite-based *N*_d_–CCN susceptibility study by Hasekamp et al.^5^. It is important to note that the OLS method tends to underestimate the slope of the fit when the predictor variable, in this case, *N*_d_, exhibits uncertainty or contains outliers^70^. Consequently, the estimates derived for *S* in this study can also be viewed as conservative lower bounds for the susceptibility. In addition to classical OLS fitting, we applied three other models to validate our results. In the classical OLS method, the fit is made on the mean of the CCN bins; in our second approach, we also tested the fit on the maximum value of each CCN bin. We also wanted to minimize the effect of potential outliers in the data by applying robust regression^71^ with iterated re-weighted least squares. This method is similar to OLS but allows different weightings for data points, enabling, for example, to downscale the effect of outliers to the fit. We applied two different weighting methods for outlying points: biweight and the Huber method, available in the MATLAB function ‘robustreg’ and introduced in more detail in ref. ^71^. All four of these methods gave comparable results for *S* (within 10% for observations and ECHAM-M7, ECHAM-SALSA and NorESM and within 35% for UKESM).

To see how accommodating the uncertainty in both *N*_d_ and CCN affects the fit and the defined *S*, we explored Bayesian errors-in-variables regression, modelling the *N*_d_ and CCN uncertainties using normal distributions on a linear scale. We treated the uncertainty standard deviations as unknown parameters and estimated them simultaneously with linear model parameters using Markov chain Monte Carlo sampling. This method gave even higher *S* values for the observations (0.881–1.070) than the OLS. *S* variabilities for model data were comparable to the OLS-based fits. Hence, the Bayesian errors-in-variables method supports the conclusions based on the OLS fits.

## Online content

Any methods, additional references, Nature Portfolio reporting summaries, source data, extended data, supplementary information, acknowledgements, peer review information; details of author contributions and competing interests; and statements of data and code availability are available at 10.1038/s41561-025-01662-y.

## Supplementary information

Supplementary Information Supplementary Information for the methods, Figs. 1–8 and Tables 1–11.

## Source data

Source Data Fig. 1 Statistical source data.

Source Data Fig. 2 Statistical source data.

Source Data Fig. 3 Statistical source data.

Source Data Fig. 4 Statistical source data.

## Data Availability

Global model data are available via the FMI METIS repository at 10.57707/fmi-b2share.6f4a6eb95fb74d81a176c7f3a92525e8. CVI data (size distributions) and meteorological data for Zeppelin are available via Stockholm University at 10.17043/zeppelin-cloud-aerosol-1. Pallas interstitial inlet data (size distributions) are available via NILU at https://ebas.nilu.no/, and Pallas Doppler lidar data are available via ACTRIS at https://cloudnet.fmi.fi/file/06fb58cc-13fc-481d-b695-287901e338ac. All Puijo data, Pallas total inlet (size distribution) and meteorological data and Zeppelin anemometer data are available via Zenodo at 10.5281/zenodo.13358529 (ref. ^72^). Source data are provided with this paper.

## Code availability

The ECHAM6–HAMMOZ code is available via the HAMMOZ consortium at https://redmine.hammoz.ethz.ch/. The availability is regulated under the HAMMOZ software licence agreement that can be downloaded from https://redmine.hammoz.ethz.ch/attachments/download/291/License_ECHAM-HAMMOZ_June2012.pdf. The ECHAM-HAMMOZ model version was echam6.3-ham2.3 The NorESM2.0.5 (10.5281/zenodo.3905091) code is available via GitHub at https://github.com/NorESMhub/NorESM.git by following the instructions given at https://noresm-docs.readthedocs.io/en/noresm2/access/releases_noresm20.html#id4. UKESM is available for use outside the Met Office through a licensing agreement (for details, see https://www.metoffice.gov.uk/research/approach/modelling-systems/unified-model).
