# Supplementary material for: High sensitivity of cloud formation to aerosol changes
Source: Nat Geosci. 2025 Apr 3;18(4):289–95. doi: 10.1038/s41561-025-01662-y (PMC11981915; doi:10.1038/s41561-025-01662-y)
Supplement: Supplementary file 1 — Supplementary Information for the methods, Figs. 1–8 and Tables 1–11. [file 41561_2025_1662_MOESM1_ESM.pdf]

nature geoscience

Supplementary information https://doi.org/10.1038/s41561-025-01662-y

High sensitivity of cloud formation to aerosol changes

In the format provided by the authors and unedited

Supplemental Information

Contents

S1 Description of measurements 2
S2 Global model descriptions 2
S2.1 Droplet activation scheme descriptions 5
S2.3 Aerosol description in ECHAM-SALSA 7
S2.4 Aerosol description in ECHAM-HAM 8
S2.5 Aerosol description in NorESM 9
S3 Map of measurement locations with CCN concentrations 11
S4 Sensitivity of the susceptibility to CCN proxy diameters 12
S5 CCN and Nd histograms for observations and models 13
S6 Cloud parcel model results 14
S7 Join-histogram and updraft analysis for Pallas station 16
S8 Join-thistogram and updraft analysis for Zeppelin station 17
S9 Median size distributions and activation curves for ECHAM-SALSA and ECHAM-HAM outputs: Pallas and Zeppelin 19
References 19

S1 Description of measurements

Table S1: Description of the measurements

Station	Coordinates	Elevation a.s.l. / above surrounding area	Environment	Data duration (aerosol data)	Measurements of Nd and CCN	Measurements updraft velocity
Puijo	62° 54' 34" N, 27° 39' 19" E	306 m/ 230 m	semi-urban	20.11.2009 – 31.12.2015	Two inlet system; interstitial and total aerosol; DMPS measurement (3-800 nm)	Lidar, 3 s resolution, data duration: 1.9.2020 - 30.11.2020
Pallas	*67°58' N, 24°07' E*	565 m /300 m	Remote, sub-Arctic	12.8.2005 - 25.12.2015	Two inlet system; interstitial and total aerosol; DMPS measurement (7-500 nm)	Lidar, 11 s time resolution, data duration: 23.8.2022 - 15.12.2023
Zeppelin	*78°54'29" N, 11°52'53" E*	474 m /474 m	Remote, Arctic	27.11.2015 – 4.2.2018	CVI; interstitial and cloud droplet residuals; DMPS measurement (10-945 nm / 5-809 nm)	Wind Anemometer, 1 s resolution, data duration: 27.11.2015 – 10.01.2017

S2 Global model descriptions

ECHAM-HAM and ECHAM-SALSA

ECHAM-HAMMOZ (ECHAM6.3-HAM2.3-MOZ1.0) is an aerosol-chemistry-climate model which couples the atmospheric model ECHAM6, aerosol model HAM, and the chemistry model MOZ. ECHAM6 is the sixth-generation atmospheric general circulation model which is the atmospheric component of Max Planck Institute for Meteorology Earth System Model (MPI-ESM). A comprehensive description of the model is given by Stevens et al.[1]. The model utilizes the spectral method to compute atmospheric circulation, incorporating a semi-Lagrangian transport scheme. In our specific model setup, the horizontal grid adopts T63 spectral truncation, complemented by 47 sigma-pressure vertical levels that conform to the terrain.

The ECHAM6 simulation employed prescribed sea surface temperatures (SSTs) and sea ice cover (SIC). Land processes were computed using the JSBACH model[2] coupled with ECHAM6. Large scale circulation is nudged towards ERA Interim reanalysis data[3].

ECHAM-HAMMOZ includes two different aerosol modules to couple aerosols to radiation and clouds, the modal scheme M7[4,5] and the sectional scheme SALSA[6]. The modal scheme HAM7 describes the size distribution as a superposition of seven lognormal modes, while SALSA uses 17 size classes to describe the size distribution. As offline emissions, Community Emissions Data System (CEDS) were used for anthropogenic emissions, BB4MIPS were used for biomass burning emissions. Mineral dust, sea salt emissions, marine dimethylsulfide, biogenic volatile organic carbon emissions were calculated online (see Tegen et al., 2019[4] for details).

Configurations of ECHAM6 for HAM7 and SALSA are otherwise identical except that they use different methods for describing size distributions, which leads to using different routines for cloud activation, aerosol microphysics, and wet deposition[7].

NorESM

The Norwegian Earth System model version 2 (Seland *et al.*[8], release 2.0.5, https://github.com/NorESMhub/NorESM) is based on the Community Earth System Model (CESM2)[9], but the ocean module is replaced by the Bergen Layered Ocean Model (BLOM, not online in these simulations), and large changes to the atmospheric component.

The atmospheric component in NorESM is named CAM6-Nor, and shares many characteristics with the Community Atmospheric model version 6 (CAM6)[10]. However, the aerosol component is replaced by OsloAero6 (details in Kirkevåg *et al.*[11] and below), and it also includes changes to the local dry and moist energy conservation, angular momentum conservation and in the computation of air-sea fluxes and deep convection. The cloud macrophysics scheme is the Cloud Layers Unified by Binomials model (CLUBB)[10], as in CAM6. Cloud microphysics in shallow convection and stratiform clouds are treated with MG2[12], which is a two-moment bulk scheme. Microphysics in deep convective clouds is based on Zhang and McFarlane[13]. The activation of cloud droplets is calculated using the Abdul-Razzak scheme[14]. Please see Bogenschutz *et al.*[3] for more information on the treatment of cloud physics in CAM6 and CAM6-Oslo.

The aerosol scheme OsloAero6 is a production-tagged aerosol and chemistry scheme which tracks sources of origin of the aerosols throughout their atmospheric lifetime. The scheme differentiates between so called “background” and “process” tracers. The background tracers determine the number of particles and an initial particle number size distribution (log-normal modes with pre-set sizes), while the process tracers alter this initial distribution and chemical composition by a look-up-table approach produced from an offline sectional scheme AeroTab[11]. The mass of each tracer is tracked and advected and in each time step, the optical properties and the size distributions for cloud activation are found using the look-up tables.

The land model is as in CESM2, the Community Land Model version 5 (CLM5)[15], and was run in BGC (bio-geo-chemistry) mode and with prognostic crop.

The simulations for this study were run with nudged meteorology (horizontal wind and surface pressure) to ERA-Interim[3] using a relaxation time of 6 h[16]. The resolution was 1.9x2.5 degrees and the model was with prescribed sea surface temperatures and sea ice data based on the Hadley Centre Sea Ice and Sea Surface Temperature data set (HADISST)[17] as described in Hurrell *et al.*[18].

UKESM1

The first version of the United Kingdom Earth System Model (UKESM1)[19] was developed using the global coupled climate model HadGEM3-GC3.1[20,21] as its physical core atmosphere-land-ocean-sea ice model. The atmospheric and land components of HadGEM3-GC3.1 consist of the Global Atmosphere 7.1 (GA7.1) configuration of the Unified Model (UM)[22] and the Global Land 7.0 configuration (GL7.0) of the Joint UK Land Environment Simulator (JULES) land surface model[22,23]. UKESM1 is coupled to additional component models that describe earth system processes encompassing marine and terrestrial biogeochemical cycles and fully interactive stratospheric–tropospheric trace gas chemistry[19]. The chemistry-aerosol component model used in UKESM to simulate atmospheric composition is the UK Chemistry and Aerosol model (UKCA)[24–27]. The aerosol scheme within UKCA is referred to as the Global Model of Aerosol Processes, GLOMAP mode[26,28,29]. This aerosol scheme employs a two-moment pseudo-modal approach and simulates multicomponent global aerosol, which includes sulphate, black carbon, organic matter and sea spray. Dust is simulated separately using the scheme of Woodward (2001)[30]. The aerosol particle size distribution is represented using five log-normal modes, nucleation soluble, Aitken soluble, accumulation soluble, coarse soluble and Aitken insoluble. GLOMAP mode includes aerosol microphysical processes of new particle formation, condensation, coagulation, wet scavenging, dry deposition and cloud processing. New particle formation from the binary homogeneous nucleation of H2SO4 and water follows Vehkamäki et al.[31] which occurs primarily in the free troposphere. New particle formation from boundary layer nucleation is not yet included[29]. The number of activated cloud droplets is simulated using the UKCA-Activate scheme[32] which uses the activation scheme of Abdul-Razzak and Ghan[14]. Cloud droplet number concentration is diagnosed from CCN and the variance of updraft velocity using the UKCA-Activate scheme of West et al.[32]. The distribution of subgrid variability of updraft velocities in UKCA-Activate is calculated according to West et al.[32] with updates as described in Mulcahy et al.[26].

Large-scale precipitation is modelled using a single-moment scheme based on Wilson and Ballard [33]. Large-scale clouds use the prognostic cloud fraction and prognostic condensate (PC2) scheme[34–36] and convection is based on the mass flux scheme of Gregory and Rowntree[37] with various extensions as documented in Gregory and Allen[38].

In this study UKESM1 is configured for Atmospheric Model Intercomparison Project (AMIP) style runs in which UKESM1 is run in its atmosphere-only configuration with AMIP-style time-evolving sea surface temperature and sea ice taken from the unmodified dataset of Durack and Taylor[39] and horizontally interpolated to the model resolution. The dynamic vegetation model[40] is replaced by prescribed vegetation properties from a coupled historical simulation with the same base model to preserve consistency in the forcing due to land use change between the UKESM1 coupled and AMIP experiments. Similarly, seawater concentrations of dimethyl sulfide (DMS) and chlorophyll-a monthly climatologies are taken from the coupled historical experiment to obtain fluxes of DMS and primary marine organic aerosol to the atmosphere[29].

External forcing datasets are consistent with the CMIP6 implementation described in Sellar et al.[41] Specifically, anthropogenic emissions, biomass-burning emissions and transient greenhouse gas concentrations are taken from Hoesly et al.[42], van Marle et al.[43] and Meinshausen et al.[44]. UKESM1 can be run at different resolutions[22] and in this study we use the N96L85 configuration, which is

1.875∘ × 1.25∘ longitude–latitude. In the vertical, the atmosphere has 85 levels up to an altitude of 85 km from the Earth's surface. These levels are divided such that there are 50 levels between 0 and 18 km and 35 levels between 18 and 85 km.

A nudged configuration was applied, where horizontal winds (but not temperature) in the model are relaxed towards fields from ERA-Interim reanalysis[45,46] following the setup design for the Aerosol Comparisons between Observations and Models (AeroCom) phase III experiment[47]. The nudging is applied between model levels 12 and 80 with a relaxation time constant of 6 hours (which is equal to the ERA-Interim reanalysis temporal resolution fields).

S2.1 Droplet activation scheme descriptions

Table S2: Droplet activation scheme in four investigated models.

GCM/ESM	Activation scheme	Reference	Any modifications from paper?	Prognostic/ Diagnostic N_d?	Lower limit for N_d (#/cm^3)
UKESM1	ARG modal	Abdul-Razzak and Ghan (2000)[14]	no	Diagnostic	5
NorESM2	ARG modal	Abdul-Razzak and Ghan (2000)[14]	no	Prognostic	none
ECHAM-SALSA	ARG sectional	Abdul-Razzak and Ghan (2002)[48]	no	Prognostic	10
ECHAM-HAM	ARG modal	Abdul-Razzak and Ghan (2000)[14]	no	Prognostic	10

Table S3. Sub-grid scale vertical velocity in four investigated models. Note: for models giving $\bar{w}$, σ_w is calculated based on Fountoukis and Nenes, 2005[49].

GCM/ESM	Approach for calculation of *w*	Activation scheme: σ_w or $\bar{w}$	Lower limit imposed for vertical velocity parameterisation	Reference
UKESM1	Sub-grid scale variability in vertical velocity is represented using the probability density function (pdf)-based approach assuming a Gaussian distribution of vertical velocities (*w*) across the grid-box with mean vertical velocity ($\bar{w}$) and standard deviation (σ_w)[26,32]. The	σ_w (20 bins)	σ_w(min)=0.01	West et al., 2014[32]

	expected number of activated droplets is calculated from the updraft pdf using 20 equally-spaced updraft bins extending out from $w = 0$ to $4\sigma_w$. σ_w is calculated following the method of Ghan et al., 1997[50]:<br><br>$\sigma_w = \max\left(\sqrt{\frac{2}{3}}\mathrm{TKE}, \sigma_{\mathrm{w(min)}}\right)$ m s^{-1}<br><br>Where TKE is the turbulent kinetic energy and σ_w(min) is a prescribed minimum value.			
NorESM2	Calculated from Cloud Layers Unified By Binormals (CLUBB) cloud scheme and average between grid cell and grid cell below.<br>Max value 10<br>Min value .2	$\bar{w}$	$\bar{w}$(min) = 0.2 ms^{-1}	Bogenschutz, P.A. *et al.* (2013)[51]
ECHAM-SALSA	TKE-based<br>Large scale vertical velocity +turbulent vertical velocity<br>wL+0.75TKE	$\bar{w}$	0	Neubauer et al., 2019 [5]
ECHAM-HAM	Same as above	Same as above	Same as above	Same as above

Table S4. Calculation of aerosol activation in four investigated models.

**GCM/ESM**	**Standard / Kappa Köhler theory**	**Method for calculation of CCN at SS_{max} from activation scheme (N_{act})**
UKESM1	Standard	Abdul-Razzak & Ghan 2000[14]
NorESM2	Standard	Abdul-Razzak & Ghan 2000[14]
ECHAM-SALSA	Standard	Abdul-Razzak & Ghan 2002[48]
ECHAM-HAM	Standard	Abdul-Razzak & Ghan 2000[14]

S2.2 Aerosol description in UKESM1

Table S5. UKCA GLOMAP-Mode aerosol scheme configuration: Properties of the aerosol size distribution in GLOMAP-Mode. Species represented are sulfate, black carbon (BC), organic matter (OM), sea salt (SS) and dust (DU).

Mode Name	Geometric mean diameter range, (nm)	Composition	Solubility	Mode width	Production
Nucleation soluble	1 - 10	SO_4, OM	Yes	1.59	Nucleation
Aitken soluble	10 - 100	SO_4, BC, OM	Yes	1.59	Growth of nucleation soluble, condensation (sol., insol. Aitken), primary SO4 emission, coagulation
Aitken insoluble	10 - 100	BC, OM	No	1.59	Primary BC/OC emission
Accumulation soluble	100 - 500	SO_4, BC, OM, SS, DU	Yes	1.40	Growth of Aitken soluble, condensation, primary SS emission, coagulation
Coarse soluble	500 - 10000	SO_4, BC, OM, SS, DU	Yes	2.0	Growth of accumulation soluble, primary SS emission

Table S6. Properties of aerosol species in UKESM1.

Species	Molar Masses (kg mol-1)	Density mass (kg m-3)	Number of dissociating ions used by UKCA-ACTIVATE	Soluble
H_2SO_4	0.098	1769.0	3	yes
BC	0.012	1500.0	0	no
OC	0.0168	1500.0	0	no
NaCl	0.05844	2165.0	2	yes
DU	0.011	2650.0	0	no
SO	0.0168	1500.0	0	no

S2.3 Aerosol description in ECHAM-SALSA

Table S7. Aerosol size distribution description in ECHAM-SALSA. Species represented are sulfate, black carbon (BC), organic carbon (OC), secondary organic aerosol (SOA), sea salt (SS) and (DU).

Mode Name	Mean radius range (nm)	Composition	Solubility	Mode width	Production
Subregion 1a	1.5 - 25	SO_4, OC, SOA	Yes	variable	Nucleation, condensation, primary SO4 and OC emissions
Subregion 2a	> 25	SO_4, OC, SOA, SS, BC, DU	Yes	variable	Growth of subregion 1a, condensation, primary SO4, OC, SS, emissions, coagulation
Subregion 2b	> 25	SO_4, OC, BC, DU	Yes	variable	Primary BC, OC, DU emissions

Table S8. Properties of aerosol species in ECHAM-SALSA

Species	Molar Masses (kg mol-1)	Density mass (kg m-3)	B term (eq. 4 in Abdul-Razzak & Ghan 2000[14])	Number of dissociating ions	Soluble
BC	0.012	2000	0	0	no
OM	0.180	1320	0.132	1	yes
3xSOA species	0.180	1320	0.132	1	yes
DU	0.250	2650	0	0	no
SO_4	0.0961	1841	0.69	2	yes
SS	0.05844	2165	1.333	2	yes

S2.4 Aerosol description in ECHAM-HAM

Table S9. Aerosol size distribution description in ECHAM-HAM. Species represented are sulfate, black carbon (BC), organic carbon (OC), secondary organic aerosol (SOA), sea salt (SS) and (DU).

Mode Name	Mean radius range (nm)	Composition	Solubility	Mode width	Production
Nucleation soluble	<5	SO_4	Yes	1.59	Nucleation
Aitken soluble	5 - 50	SO_4, BC, OC	Yes	1.59	Growth of nucleation soluble, condensation, transfer from Aitken insoluble, primary SO4 and OC emissions, coagulation
Aitken insoluble	5 - 50	BC, OC	No	1.59	Primary BC/OC emission

Accumulation soluble	50 - 500	SO_4, BC, OC, SS, DU	Yes	1.59	Growth of Aitken soluble, condensation, transfer from Accumulation soluble, primary SS, SO4, OC emissions, coagulation
Accumulation insoluble	50 - 500	SO_4, BC, DU	No	1.59	Growth of Aitken insoluble, condensation, primary DU emissions, coagulation
Coarse soluble	> 500	SO_4, BC, OC, SS, DU	Yes	2.0	Growth of accumulation soluble, transfer from Coarse insoluble, primary SS emissions, condensation, coagulation
Coarse insoluble	> 500	SO_4, BC, DU	No	2.0	Growth of accumulation insoluble, primary DU emissions, condensation, coagulation

Table S10. Properties of aerosol species in ECHAM-HAM. Species represented are sulfate, black carbon (BC), organic matter (OM), sea salt (SS) and (DU).

Species	Molar Masses (kg mol-1)	Density mass (kg m-3)	B term (eq. 4 in Abdul-Razzak & Ghan 2000[14])	Number of dissociating ions	Soluble
BC	0.012	2000	0	0	no
OM	0.180	2000	0.2	1	yes
DU	0.250	2650	0	0	no
SO_4	0.0961	1841	0.69	2	yes
SS	0.05844	2165	1.333	2	yes

S2.5 Aerosol description in NorESM

The aerosol scheme OsloAero6 is a production-tagged aerosol and chemistry scheme which tracks sources of origin of the aerosols throughout their atmospheric lifetime. The scheme differentiates between so-called "background" and "process" tracers (see e.g. Table 2 in Kirkevåg *et al.*, 2018[11]). The background tracers determine the number of particles and an initial particle number size distribution (log-normal modes with pre-set sizes), while the process tracers alter this initial distribution and chemical composition by a look-up-table approach produced from an offline sectional scheme AeroTab[11]. The mass of each tracer is tracked and advected and in each time step, the optical properties and the size distributions for cloud activation are found using the look-up tables. The modes used in the activation are the log-normal fits after the AeroTab lookup table approach is called and the new sigma is extracted. Finally the NMR is calculated.

Table S11. Properties of aerosol species in NorESM

Species	Molar Masses (kg mol-1)	Density mass (kg m-3)	B term (eq. 4 in ARG 2000)	Number of dissociating ions
Ammonium sulphate (SO_4)	0.132	1769	0.507	3
BC	0.012	1800	5e-7	1
OM (OM and SOA)	0.1682	1500	0.14	1
Mineral dust (DST)	135	2600	0.069	2
Sea salt (SS)	0.05844	2200	1.20	2

S3 Map of measurement locations with CCN concentrations

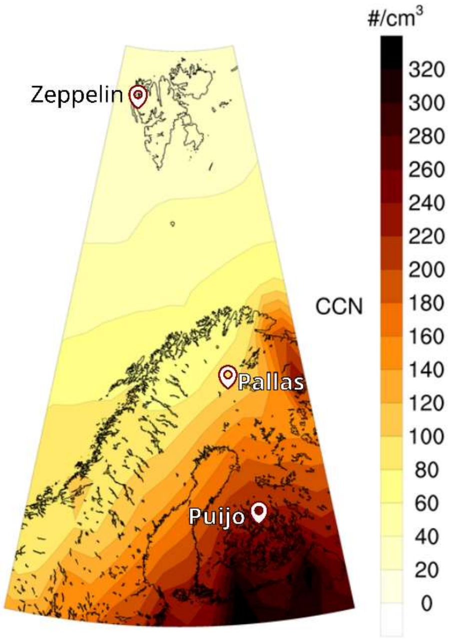

Figure S1. A map showing the location of measurement sites and CCN concentration from ECHAM-HAM.

S4 Sensitivity of the susceptibility to CCN proxy diameters

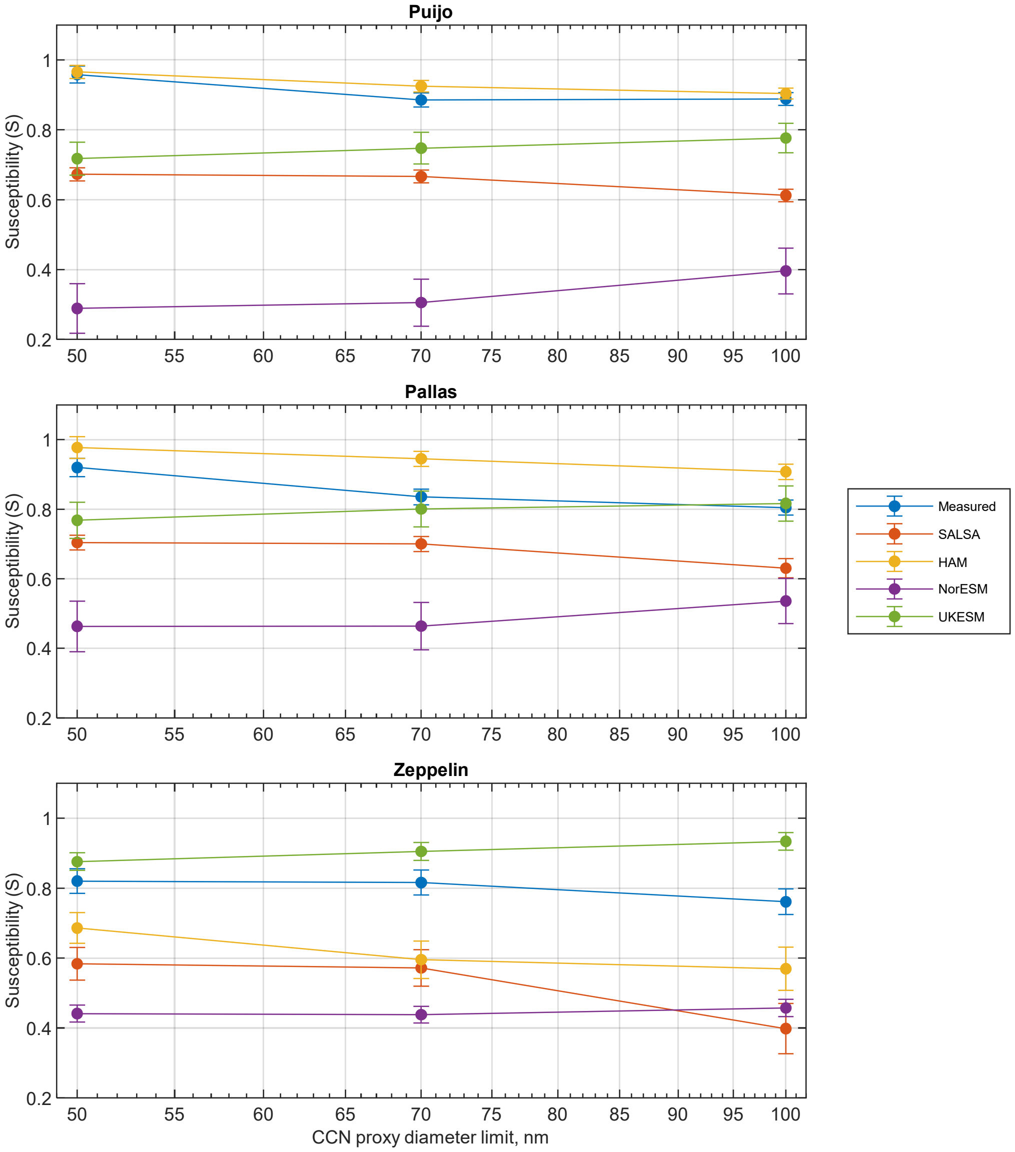

Figure S2: Fitted value for the susceptibility with different size limits for CCN proxy (error bars indicate standard deviation of the results). Error bars represent 95% confidence bounds and number of data points (N) for each fit is given in Figures 3, S5 and S6.

S5 CCN and Nd histograms for observations and models

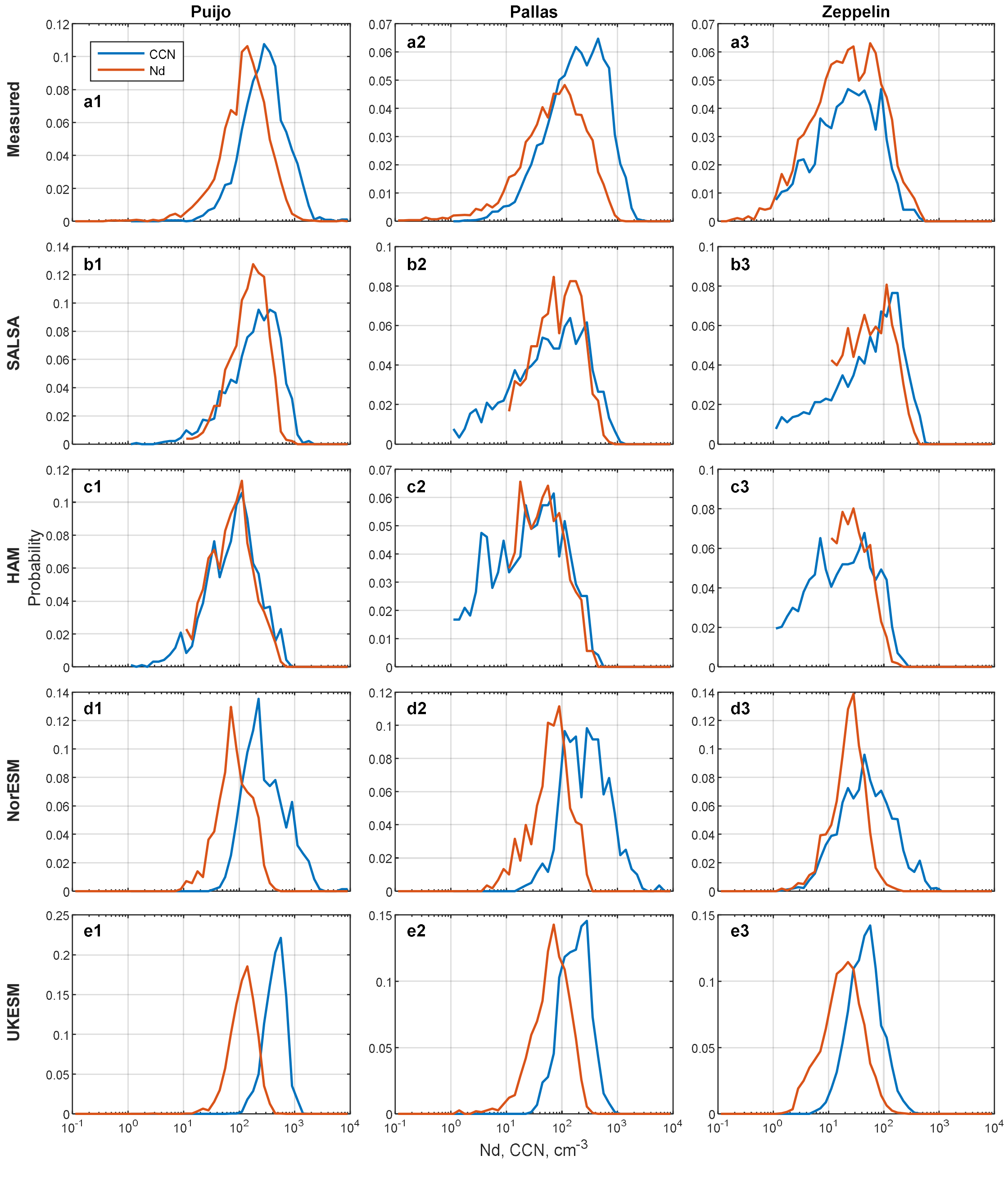

Figure S3. CCN and Nd pdfs for Puijo (column 1), Pallas (column 2) and Zeppelin (column 3). Observations are shown in a row **a** and different models at rows **b-e**.

S6 Cloud parcel model results

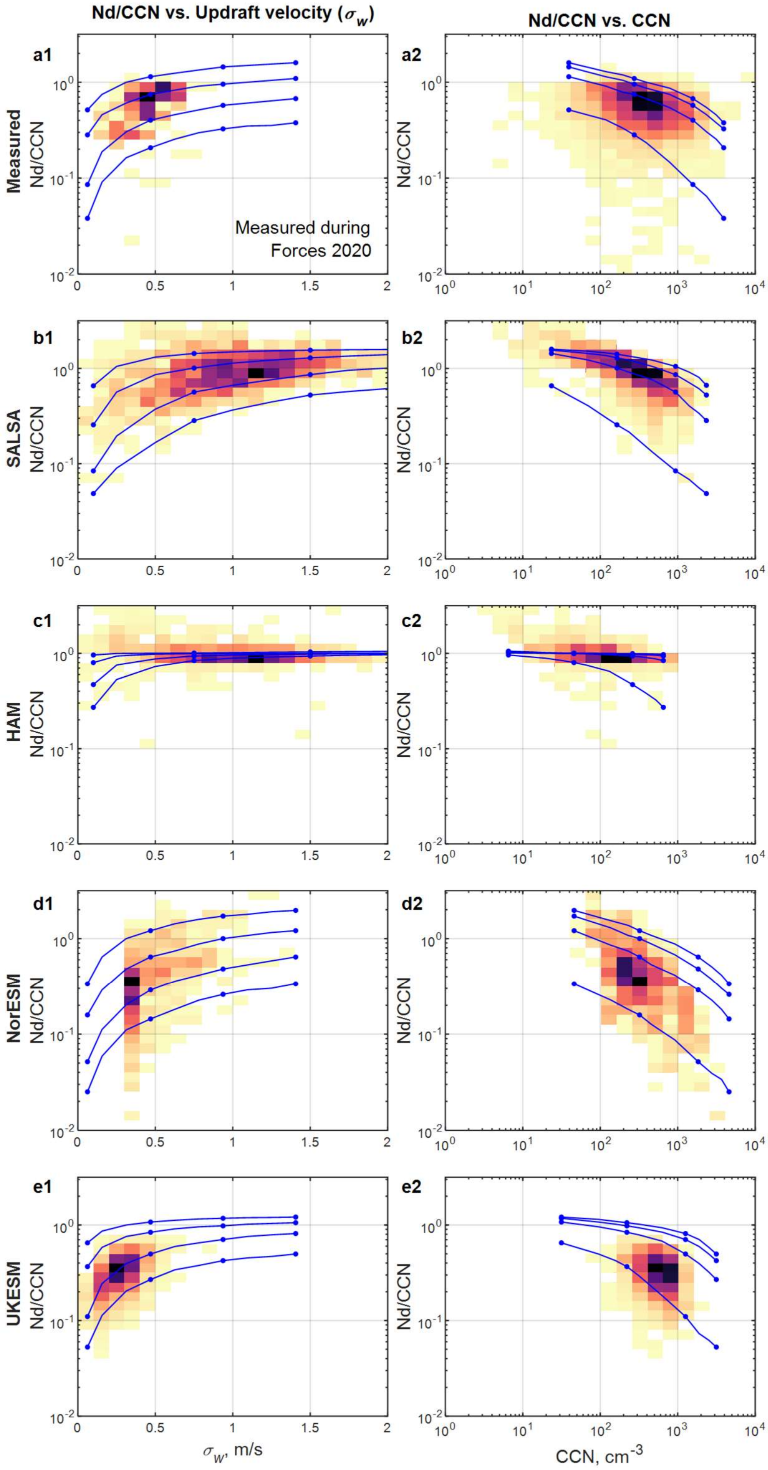

Figure S4 Cloud parcel model derived theoretical estimates for observations (panels **a1-a2**) and models (panels **b1-e2**) of activated cloud droplet concentrations for different updraft velocities and aerosol concentrations. As an input for parcel model the mean aerosol size distribution is scaled with constant factors from 0.1 to 10 to derive different

CCN concentrations and the updraft velocity is selected to cover the corresponding values form observations or models. For simplicity composition and temperature are kept as constant. The agreement between the theoretical lines and the model/observational data confirms that the activation of cloud droplets is the main driver of the CCN-CDNC-w relationship. Here the disagreement for example in the case of NorESM might be due to changing shape of aerosol size distribution as a function of CCN concentration, since we use the average aerosol properties as input to the parcel model.

S7 Join-histogram and updraft analysis for Pallas station

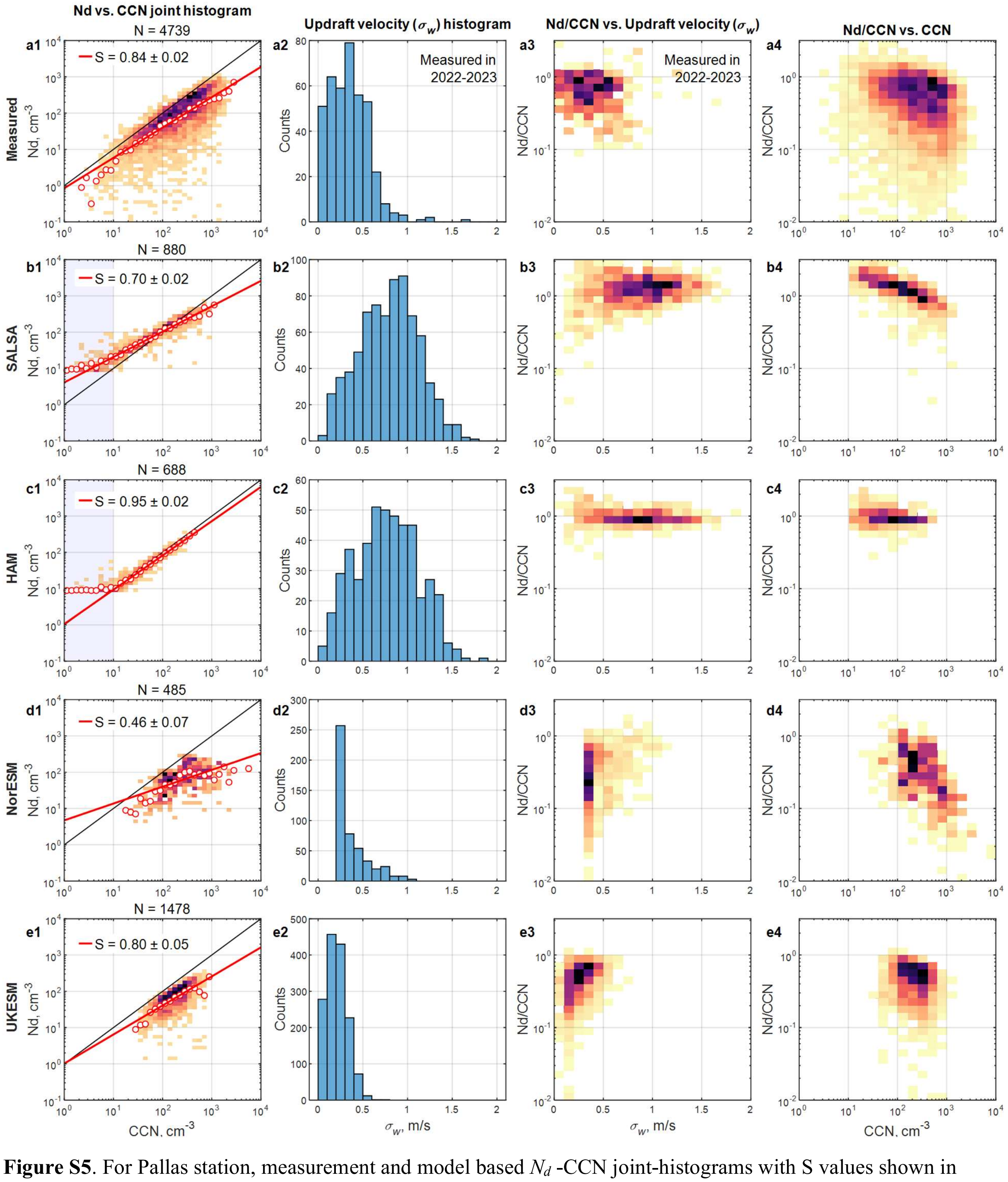

Figure S5. For Pallas station, measurement and model based N_d -CCN joint-histograms with S values shown in panels **a1-e1** (dot color indicates probability (blue=low, red=high), red edged circles are N_d mean values of at certain CCN bin), and the modeled pdfs for σ_w shown in panels **a2-e2**. The ratio of N_d to CCN is plotted as a function of σ_w (panels **a3-e3**) and CCN concentration (panels **a4-e4**) (color indicates probability intensity: orange=low, black=high). The data points from the shaded area of figures b1 and c1 are omitted in the analysis showed in panels b3,c3,b4,c4.

When compared to Puijo data, there is substantial scatter in measured N_d -CCN joint-histogram in Pallas as can be seen in Fig. S3. a). Some of the scatter and especially the low N_d values in the N_d -CCN plot can be caused by foggy conditions where activation is known to be weaker than in clouds[52]. Although both Puijo and Pallas stations are located on top of the hill, Puijo observations are conducted in a 75m tower.

S8 Join-thistogram and updraft analysis for Zeppelin station

Note that in Zeppelin, the orography significantly affects the updrafts measured in the station. This can be seen in Fig. S6 a2, where the significant shift of σ_w to lower values can be seen when the investigation is limited to low wind speed (≤ 1 m/s). This is because at low wind speeds the effect of orography to σ_w is negligible. Hence, when modeled σ_w is compared to observations, the comparison should be done using the observations with wind speed limit of ≤ 1 m/s as we want to compare the observations to modeled updrafts representing larger grid box area.

Worth mentioning is the special characteristic seen with both ECHAM versions related to σ_w, which gets an increasing amount of values very close to 0m/s when the analysis is conducted higher in the boundary layer as done in Zeppelin[53]. Due to this activation is more often in the updraft limited region, N_d gets very small values and susceptibility is decreased.

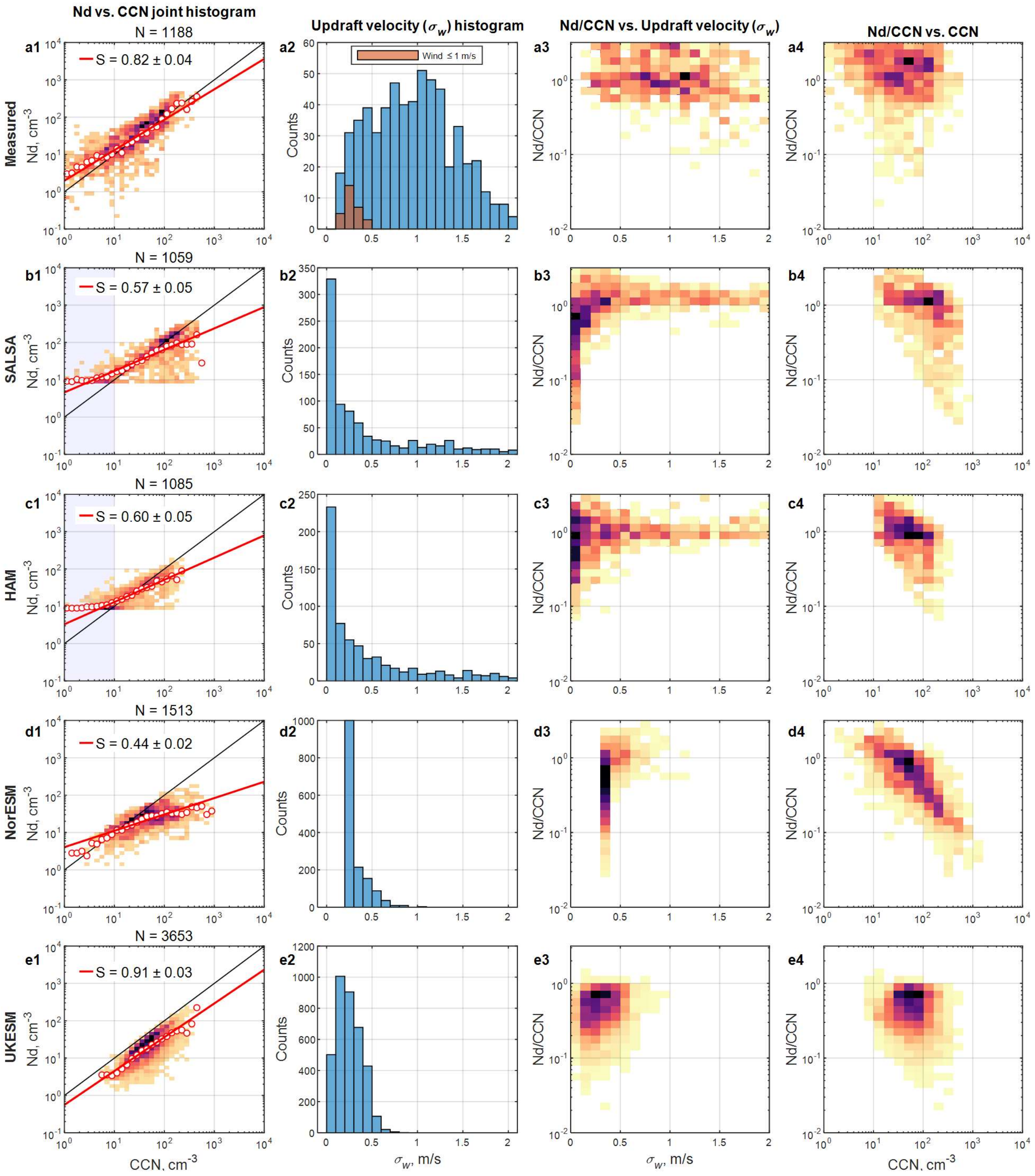

Figure S6. For Zeppelin station, measurement and model based N_d -CCN joint-histograms with S values shown in panels **a1-e1** (dot color indicates probability (blue low, red high), red edged circles are N_d mean values of at certain CCN bin), and the measured and modeled pdfs for σ_w shown in panels **a2-e2**. The ratio of N_d to CCN is plotted as a function of σ_w (panels **a3-e3**) and CCN concentration (panels **a4-e4**) (color indicates probability intensity: orange=low, black=high). In **a2**, σ_w is also plotted with wind velocity of 1 m/s and lower in brown color. The data points from the shaded area of figures b1 and c1 are omitted in the analysis showed in panels b3,c3,b4,c4.

S9 Median size distributions and activation curves for ECHAM-SALSA and ECHAM-HAM outputs: Pallas and Zeppelin

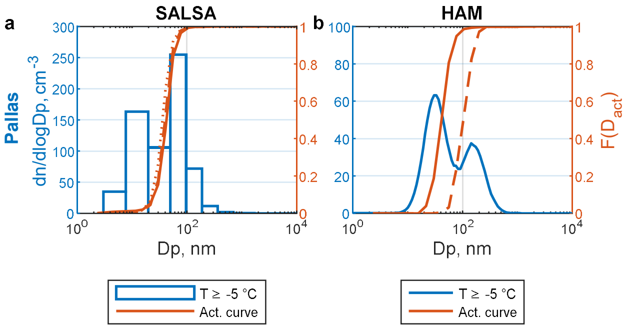

Figure S7. Median size distributions and median activation curves for two ECHAM model versions (ECHAM-SALSA in **a** and ECHAM-HAM in **b** for Pallas location. In **a**, the activation curves of three size bins (bins 3-5) are shown and in **b** the activation curve for accumulation mode (solid line) and Aitken mode (dashed line) are shown.

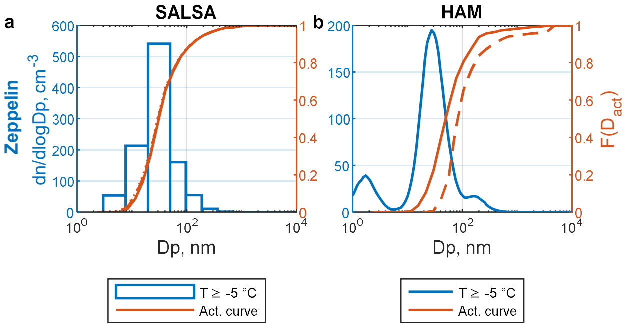

Figure S8. Median size distributions and median activation curves for two ECHAM model versions (ECHAM-SALSA in **a** and ECHAM-HAM in **b** for Zeppelin location. In **a**, the activation curves of three size bins (bins 3-5) are shown and in **b** the activation curve for accumulation mode (solid line) and Aitken mode (dashed line) are shown.

References

1. Stevens, B. *et al.* Atmospheric component of the MPI-M Earth System Model: ECHAM6. *J. Adv. Model. Earth Syst.* **5**, 146–172 (2013).

2. Raddatz, T. J. *et al.* Will the tropical land biosphere dominate the climate–carbon cycle feedback during the twenty-first century? *Clim. Dyn.* **29**, 565–574 (2007).

3. Berrisford, P. *et al.* Atmospheric conservation properties in ERA-Interim. *Q. J. R. Meteorol. Soc.* **137**, 1381–1399 (2011).

4. Tegen, I. *et al.* The global aerosol–climate model ECHAM6.3–HAM2.3 – Part 1: Aerosol evaluation. *Geosci. Model Dev.* **12**, 1643–1677 (2019).

5. Neubauer, D. *et al.* The global aerosol–climate model ECHAM6.3–HAM2.3 – Part 2: Cloud evaluation, aerosol radiative forcing, and climate sensitivity. *Geosci. Model Dev.* **12**, 3609–3639 (2019).

6. Kokkola, H. *et al.* SALSA2.0: The sectional aerosol module of the aerosol–chemistry–climate model ECHAM6.3.0-HAM2.3-MOZ1.0. *Geosci. Model Dev.* **11**, 3833–3863 (2018).

7. Holopainen, E., Kokkola, H., Laakso, A. & Kühn, T. In-cloud scavenging scheme for sectional aerosol modules – implementation in the framework of the Sectional Aerosol module for Large Scale Applications version 2.0 (SALSA2.0) global aerosol module. *Geosci. Model Dev.* **13**, 6215–6235 (2020).

8. Seland, Ø. *et al.* Overview of the Norwegian Earth System Model (NorESM2) and key climate response of CMIP6 DECK, historical, and scenario simulations. *Geosci. Model Dev.* **13**, 6165–6200 (2020).

9. Danabasoglu, G. *et al.* The Community Earth System Model Version 2 (CESM2). *J. Adv. Model. Earth Syst.* **12**, e2019MS001916 (2020).

10. Bogenschutz, P. A. *et al.* The path to CAM6: coupled simulations with CAM5.4 and CAM5.5. *Geosci. Model Dev.* **11**, 235–255 (2018).

11. Kirkevåg, A. *et al.* A production-tagged aerosol module for Earth system models, OsloAero5.3 – extensions and updates for CAM5.3-Oslo. *Geosci. Model Dev.* **11**, 3945–3982 (2018).

12. Gettelman, A. & Morrison, H. Advanced Two-Moment Bulk Microphysics for Global Models. Part I: Off-Line Tests and Comparison with Other Schemes. *J. Clim.* **28**, 1268–1287 (2015).

13. Zhang, G. J. & McFarlane, N. A. Sensitivity of climate simulations to the parameterization of cumulus convection in the Canadian climate centre general circulation model. *Atmosphere-Ocean* **33**, 407–446 (1995).

14. Abdul-Razzak, H. & Ghan, S. J. A parameterization of aerosol activation: 2. Multiple aerosol types. *J. Geophys. Res. Atmospheres* **105**, 6837–6844 (2000).

15. Lawrence, D. M. *et al.* The Community Land Model Version 5: Description of New Features, Benchmarking, and Impact of Forcing Uncertainty. *J. Adv. Model. Earth Syst.* **11**, 4245–4287 (2019).

16. Kooperman, G. J. *et al.* Constraining the influence of natural variability to improve estimates of global aerosol indirect effects in a nudged version of the Community Atmosphere Model 5. *J. Geophys. Res. Atmospheres* **117**, 2012JD018588 (2012).

17. Rayner, N. A. *et al.* Global analyses of sea surface temperature, sea ice, and night marine air temperature since the late nineteenth century. *J. Geophys. Res. Atmospheres* **108**, 2002JD002670 (2003).

18. Hurrell, J. W., Hack, J. J., Shea, D., Caron, J. M. & Rosinski, J. A New Sea Surface Temperature and Sea Ice Boundary Dataset for the Community Atmosphere Model. *J. Clim.* **21**, 5145–5153 (2008).

19. Sellar, A. A. *et al.* UKESM1: Description and Evaluation of the U.K. Earth System Model. *J. Adv. Model. Earth Syst.* **11**, 4513–4558 (2019).

20. Kuhlbrodt, T. *et al.* The Low-Resolution Version of HadGEM3 GC3.1: Development and Evaluation for Global Climate. *J. Adv. Model. Earth Syst.* **10**, 2865–2888 (2018).

21. Williams, K. D. *et al.* The Met Office Global Coupled Model 3.0 and 3.1 (GC3.0 and GC3.1) Configurations. *J. Adv. Model. Earth Syst.* **10**, 357–380 (2018).

22. Walters, D. *et al.* The Met Office Unified Model Global Atmosphere 6.0/6.1 and JULES Global Land 6.0/6.1 configurations. *Geosci. Model Dev.* **10**, 1487–1520 (2017).

23. Best, M. J. *et al.* The Joint UK Land Environment Simulator (JULES), model description – Part 1: Energy and water fluxes. *Geosci. Model Dev.* **4**, 677–699 (2011).

24. Morgenstern, O. *et al.* Evaluation of the new UKCA climate-composition model – Part 1: The stratosphere. *Geosci. Model Dev.* **2**, 43–57 (2009).

25. O'Connor, F. M. *et al.* Evaluation of the new UKCA climate-composition model – Part 2: The Troposphere. *Geosci. Model Dev.* **7**, 41–91 (2014).

26. Mulcahy, J. P. *et al.* Improved Aerosol Processes and Effective Radiative Forcing in HadGEM3 and UKESM1. *J. Adv. Model. Earth Syst.* **10**, 2786–2805 (2018).

27. Archibald, A. T. *et al.* Description and evaluation of the UKCA stratosphere–troposphere chemistry scheme (StratTrop vn 1.0) implemented in UKESM1. *Geosci. Model Dev.* **13**, 1223–1266 (2020).

28. Mann, G. W. *et al.* Description and evaluation of GLOMAP-mode: a modal global aerosol microphysics model for the UKCA composition-climate model. *Geosci. Model Dev.* **3**, 519–551 (2010).

29. Mulcahy, J. P. *et al.* Description and evaluation of aerosol in UKESM1 and HadGEM3-GC3.1 CMIP6 historical simulations. *Geosci. Model Dev.* **13**, 6383–6423 (2020).

30. Woodward, S. Modeling the atmospheric life cycle and radiative impact of mineral dust in the Hadley Centre climate model. *J. Geophys. Res. Atmospheres* **106**, 18155–18166 (2001).

31. Vehkamäki, H. *et al.* An improved parameterization for sulfuric acid–water nucleation rates for tropospheric and stratospheric conditions. *J. Geophys. Res. Atmospheres* **107**, (2002).

32. West, R. E. L. *et al.* The importance of vertical velocity variability for estimates of the indirect aerosol effects. *Atmospheric Chem. Phys.* **14**, 6369–6393 (2014).

33. Wilson, D. R. & Ballard, S. P. A microphysically based precipitation scheme for the UK meteorological office unified model. *Q. J. R. Meteorol. Soc.* **125**, 1607–1636 (1999).

34. Wilson, D. R., Bushell, A. C., Kerr-Munslow, A. M., Price, J. D. & Morcrette, C. J. PC2: A prognostic cloud fraction and condensation scheme. I: Scheme description. *Q. J. R. Meteorol. Soc.* **134**, 2093–2107 (2008).

35. Wilson, D. R. *et al.* PC2: A prognostic cloud fraction and condensation scheme. II: Climate model simulations. *Q. J. R. Meteorol. Soc.* **134**, 2109–2125 (2008).

36. Morcrette, C. J. Improvements to a prognostic cloud scheme through changes to its cloud erosion parametrization. *Atmospheric Sci. Lett.* **13**, 95–102 (2012).

37. Gregory, D. & Rowntree, P. R. A Mass Flux Convection Scheme with Representation of Cloud Ensemble Characteristics and Stability-Dependent Closure. *Mon. Weather Rev.* **118**, 1483–1506 (1990).

38. Gregory, D. The effect of convective scale downdraughts upon NWP and climate simulations. in *9th conference on numerical weather prediction* 122–123 (Amer. Meteor. Soc., 1991).

39. Durack, P. J. & Taylor, K. E. PCMDI AMIP SST and sea-ice boundary conditions version 1.1.3. [object Object] https://doi.org/10.22033/ESGF/INPUT4MIPS.1735 (2017).

40. Cox, P. M. Description of the "TRIFFID" dynamic global vegetation model, Hadley Centre Technical Note, met Office Hadley Centre, Exeter, Devon, United Kingdom. (2001).

41. Sellar, A. A. *et al.* Implementation of U.K. Earth System Models for CMIP6. *J. Adv. Model. Earth Syst.* **12**, e2019MS001946 (2020).

42. Hoesly, R. M. *et al.* Historical (1750–2014) anthropogenic emissions of reactive gases and aerosols from the Community Emissions Data System (CEDS). *Geosci. Model Dev.* **11**, 369–408 (2018).

43. Van Marle, M. J. E. *et al.* Historic global biomass burning emissions for CMIP6 (BB4CMIP) based on merging satellite observations with proxies and fire models (1750–2015). *Geosci. Model Dev.* **10**, 3329–3357 (2017).

44. Meinshausen, M. *et al.* Historical greenhouse gas concentrations for climate modelling (CMIP6). *Geosci. Model Dev.* **10**, 2057–2116 (2017).

45. Telford, P. J., Braesicke, P., Morgenstern, O. & Pyle, J. A. Technical Note: Description and assessment of a nudged version of the new dynamics Unified Model. *Atmospheric Chem. Phys.* **8**, 1701–1712 (2008).

46. Dee, D. P. *et al.* The ERA-Interim reanalysis: configuration and performance of the data assimilation system. *Q. J. R. Meteorol. Soc.* **137**, 553–597 (2011).

47. Kim, P., Partridge, D. & Haywood, J. Constraining the model representation of the aerosol life cycle in relation to sources and sinks. https://meetingorganizer.copernicus.org/EGU2020/EGU2020-21948.html (2020) doi:10.5194/egusphere-egu2020-21948.

48. Abdul-Razzak, H. & Ghan, S. J. A parameterization of aerosol activation 3. Sectional representation. *J. Geophys. Res. Atmospheres* **107**, (2002).

49. Fountoukis, C. & Nenes, A. Continued development of a cloud droplet formation parameterization for global climate models. *J. Geophys. Res. Atmospheres* **110**, 2004JD005591 (2005).

50. Ghan, S. J., Leung, L.-Y., Easter, R. C. & Abdul-Razzak, H. Prediction of Cloud Droplet Number in a General Circulation Model. *J. Geophys. Res. Atmospheres* **102**, (1997).

51. Bogenschutz, P. A. *et al.* Higher-Order Turbulence Closure and Its Impact on Climate Simulations in the Community Atmosphere Model. *J. Clim.* **26**, 9655–9676 (2013).

52. Hammer, E. *et al.* Size-dependent particle activation properties in fog during the ParisFog 2012/13 field campaign. *Atmospheric Chem. Phys.* **14**, 10517–10533 (2014).

53. Nordling, K. *et al.* Technical note: Emulation of a large-eddy simulator for stratocumulus clouds in a general circulation model. *Atmospheric Chem. Phys.* **24**, 869–890 (2024).
